# Metabolic Responses of Dietary Fiber during Heat Stress: Effects on Reproductive Performance and Stress Level of Gestating Sows

**DOI:** 10.3390/metabo12040280

**Published:** 2022-03-23

**Authors:** SeungMin Oh, Abdolreza Hosseindoust, SangHun Ha, Joseph Moturi, JunYoung Mun, Habeeb Tajudeen, JinSoo Kim

**Affiliations:** 1 Gyeongbuk Livestock Research Institute, Yeongju 63052, Korea; ssamgoon@korea.kr; 2 Department of Animal Industry Convergence, Kangwon National University, Chuncheon 24341, Korea; hosseindoust@kangwon.ac.kr (A.H.); kayne7602@kangwon.ac.kr (S.H.); motkondo@gmail.com (J.M.); 202016455@kangwon.ac.kr (J.M.); habeebtop@gmail.com (H.T.)

**Keywords:** metabolites, metabolomics, short-chain fatty acid, digestibility, fermentation, intestine

## Abstract

Heat stress is an important issue, and the addition of fiber to the diet is an option in modifying intestinal health. This study evaluated the effect of acid detergent fiber (ADF) levels on reproductive performance, intestinal integrity, and metabolism of gestating sows, and its carry-over effect on the lactation period during heat stress. The diets included 4.3% (Low fiber; LF), 5.4% (Medium fiber; MF), and 6.5% (High fiber; HF) ADF. Sows fed the HF diet showed a lower respiratory rate, hair cortisol concentration, and farrowing duration compared with the LF treatment. The HF diet increased the pyruvate, citrate cycle, glyoxylate, dicarboxylate, and thiamine metabolism compared with the MF. The concentration of acetate and total short-chain fatty acids were increased in the sows fed the HF diet. The gene expression of glucose transporter 3 and glucose transporter 4 was increased in the HF treatment. The gene expression of heat shock protein 70 was decreased in the HF treatment. The HF diet during gestation increased feed intake, constipation index, piglet weight, and litter weight compared with the LF. Sows in the LF treatment showed the greatest digestibility of crude protein and the lowest digestibility of ADF. In conclusion, a 6.5% ADF level is recommended for gestating sows during heat stress.

## 1. Introduction

Over the past two decades, heat stress has become a major concern in the animal production industry due to global warming. Besides global warming, breeding programs have resulted in improved reproductive performance, but, in turn, increased their susceptibility to stressors. Heat stress elicits metabolic, behavioral, and physiological changes including increased respiration, and decreased feed intake at the expense of reduced milk production [1,2,3]. The decreased feed intake and metabolic rate is a natural attempt to reduce metabolic heat production [4,5]. Reduction of voluntary feed intake in the lactating sow results in reduced milk production and thus reduced reproductive performance [2,6]. In addition to the influence on feed consumption, a high ambient temperature may affect the fecal metabolites concentration of heat-stressed lactating sows.

Proper protein and energy feeding has been always a challenge to optimize the performance of farm animals during heat stress [6,7]. During heat stress, the decrease of dietary acid detergent fiber (ADF) and protein has been a logical procedure to encourage feed intake and decrease dietary heat increment [8]. On the other hand, a change in feed composition from low-fiber to high-fiber has been associated with improved intestinal integrity [9,10], nutrient digestibility [11], colostrum fat content [11,12], fecal score [12,13], and welfare [14]. Dietary fibers are subjected to bacterial fermentation and production of short-chain fatty acid (SCFA), which increases intestinal health [3,13,15]. The improved intestinal integrity during gestation may be reflected during lactation, with greater potential for digestibility of nutrients, and possibly more efficient digestion. In particular because the feed intake of lactating sows is between two to three times higher than gestating sows, and even a small increase in dietary fiber level may induce further stress and compromise litter performance [16,17]. During gestation the feed intake is limited and it significantly decreases the heat production during digestion. However, little is known about the amount of dietary fiber and its effect on heat-stressed gestating sows. Regarding the importance of fiber to improve intestinal integrity and increase satiety in gestating sows, it seems necessary to understand how much dietary fiber can be adequate during high ambient temperatures. In the current study, gestating sows were fed with different ADF concentrations to evaluate the influences of fiber on reproductive performance, digestibility of nutrients, the stress level, and the concentration of metabolites in the intestine.

## 2. Results

### 2.1. Sow Performance

Dietary ADF level did not affect the body weight (BW) and backfat thickness either at days 90 and 112 of gestation, or at weaning (Table 1). The HF diet increased (*p* < 0.01) the feed intake of sows compared with the LF diet, however, there was no difference between the MF, LF, or HF treatments. The HF diet reduced (*p* < 0.01) the farrowing duration of sows compared with the MF and LF diets. Moreover, dietary supplementation with MF decreased (*p* < 0.01) farrowing duration compared with the LF diet. The HF diet increased (*p* < 0.01) the constipation index compared with the MF and LF diets. The MF diet showed a higher (*p* < 0.01) constipation index in sows rather than the LF diet. There was no change in the weaning to estrus interval among the treatments. The ADF level of diet did not affect the total born, weaned piglets, and mortality (Table 2). Sows in the HF treatment showed a greater (*p* < 0.01) piglet weight and litter weight compared with the LF treatment at weaning, however, no difference was observed between the HF and MF treatments.

### 2.2. Nutrient Digestibility

Digestibility of dry matter (DM) and gross energy (GE) were not affected by dietary ADF (Table 3). Sows in the LF treatment showed the highest (*p* < 0.01) digestibility of crude protein (CP). In addition, a greater (*p* < 0.01) digestibility of CP was observed in the sows fed the MF diet rather than HF diet. There was no change in the digestibility of neutral detergent fiber (NDF), however, the greatest (*p* < 0.01) digestibility of ADF was observed in sows that were fed HF diet. Sows in the MF treatment showed a higher (*p* < 0.01) digestibility of ADF compared with the LF treatment.

### 2.3. Heat Stress Factors, and Plasma Insulin and Glucose

**Figure 1:**
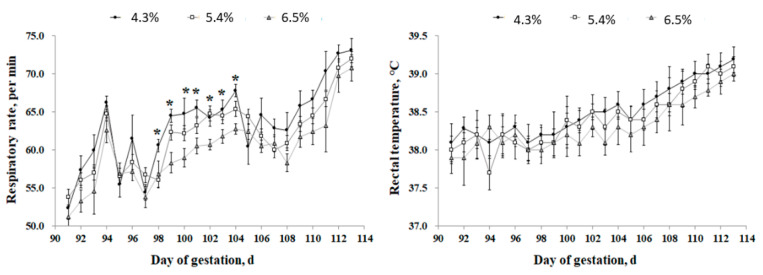
Effect of dietary acid detergent fiber levels in gestation diets on respiratory rate and rectal temperature of sows from day 90 to 114 under high ambient temperatures. Values represent means ± standard error. Asterisks (*) indicate statistical significance (*p* < 0.05).

**Figure 2:**
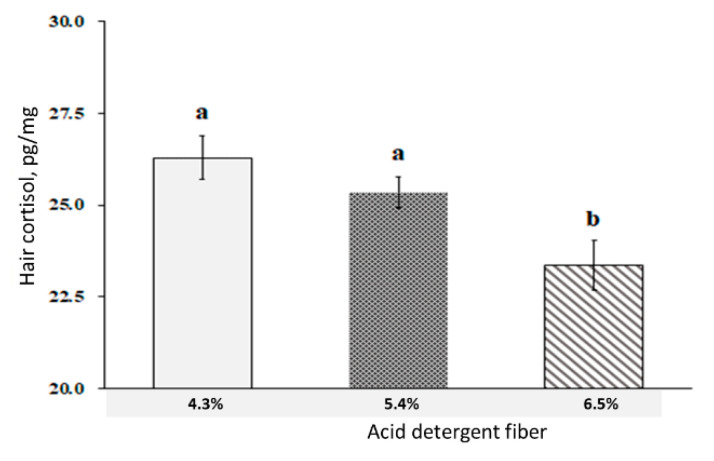
Effect of dietary acid detergent fiber levels in gestation diets on hair cortisol accumulation of sows from day 90 to 112 under high ambient temperature. Values represent means ± standard error. a,b means with different superscripts on the bar differ significantly (*p* < 0.05).

**Figure 3:**
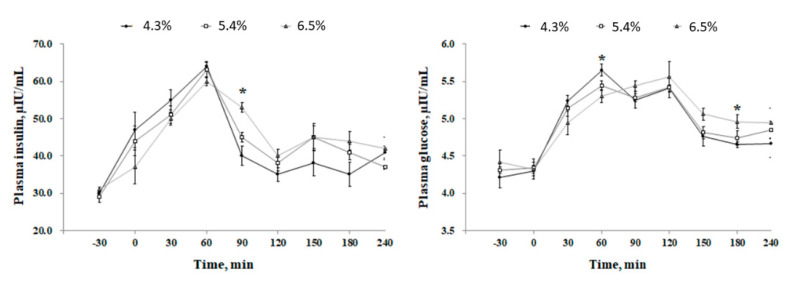
Effect of dietary acid detergent fiber levels in gestation diets on plasma insulin and glucose (d 112). Values represent means ± standard error. Asterisks (*) indicate statistical significance (*p* < 0.05).

Sows fed the low fiber (LF) diet showed a higher (*p* < 0.05) respiratory rate at days 98; 100; 102; and 104 compared with the medium fiber (MF) and high fiber (HF) treatments, however, there was no difference between the treatments at days 90–97 and 105–114 (Figure 1). There was no respiratory rate difference between the MF and HF diets throughout the experiment. There was no change in rectal temperature among the treatments. The results in Figure 2 showed that the hair cortisol concentration of sows was lower (*p* < 0.05) in the HF treatment compared with the MF and LF treatments. There was no hair cortisol difference between the HF and MF treatments. Blood insulin level was increased at min 90 after the meal in the HF diet compared with the MF and LF treatments (Figure 3), however, there was no difference in blood insulin between the MF and LF treatments. The dietary ADF level did not affect the insulin level at other times. The blood glucose level was increased (*p* < 0.05) in the LF treatment at min 60 after the meal but decreased (*p* < 0.01) at min 180 compared with the HF treatment. However, the dietary ADF level did not affect the glucose level at other sampling times. There was no difference in blood glucose between the MF and LF treatments.

### 2.4. Metabolites

**Figure 4:**
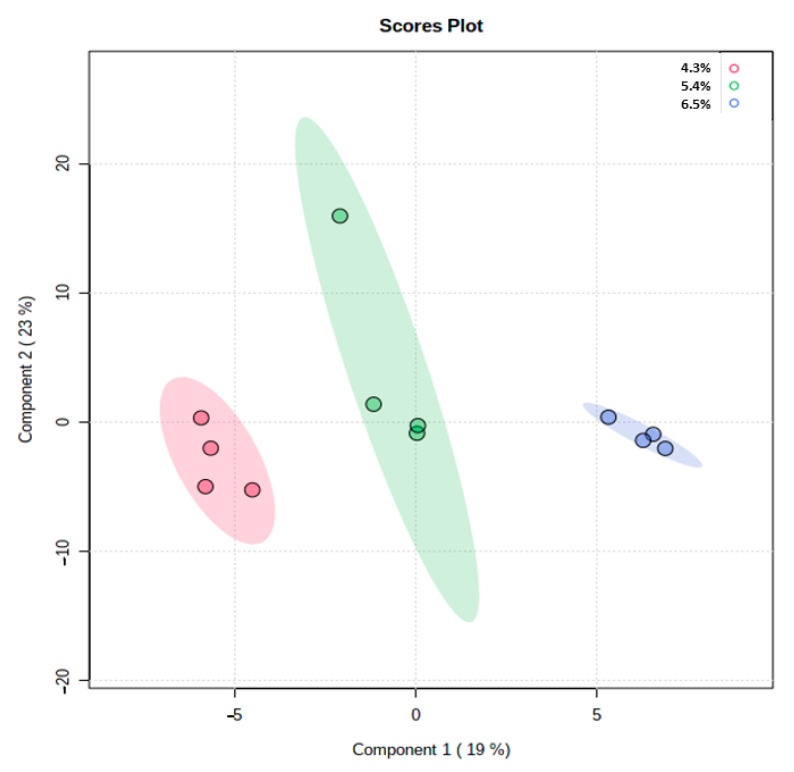
Partial least squares projection to latent structures and discriminant analysis based on the fecal compounds’ data among acid detergent fiber treatments.

**Figure 5:**
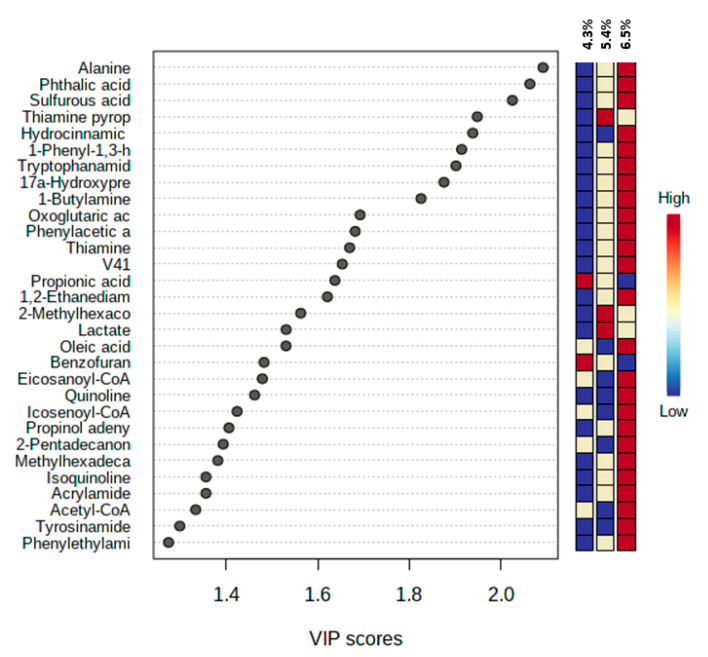
Top 30 Significant compounds. Metabolites accountable for class discrimination with variable importance projection (VIP) > 1 among acid detergent fiber treatments.

**Figure 6:**
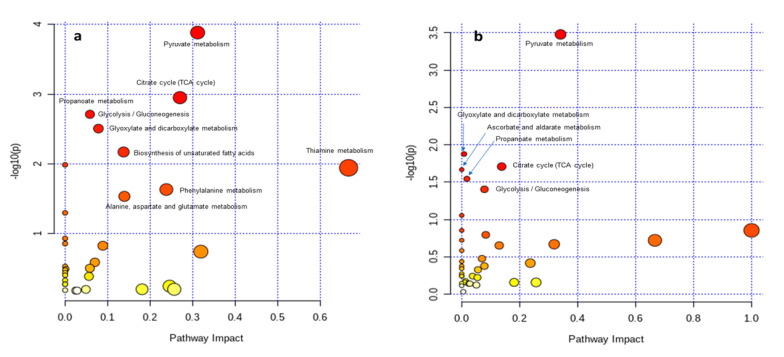
Metabolome view map of the differential metabolites (variable importance projection > 1, *p* < 0.05) identified in the feces of sows fed different acid detergent fiber levels ((**a**): 4.3% vs. 6.5%; (**b**): 5.4% vs. 6.5%) during late gestation. The x-axis represents the pathway impact and the y-axis represents the pathway enrichment. The node color is based on its *p*-value, and the node radius is determined based on the pathway impact values. Larger sizes and darker colors represent higher pathway enrichment and impact values, respectively.

The partial least squares-discriminant analysis results indicated that there were variations in the metabolites based on the different ADF groups (Figure 4). The variable importance projection (VIP) > 1 and *p* < 0.05 were applied to identify the effects of the compound on the variations. The metabolites, including carbohydrates; fatty acids; amino acids; lipids; and organic acids, were detected in multiple biochemical processes in the feces of the sow. The changes in metabolites are shown in Figure 5. The levels of alanine; phthalic acid; sulfurous acid; hydrocinnamic acid; 1-phenyl-1,3-h; tryptophanamide; 17α-hydroxypregnenolone; 1-Butylamine; oxoglutaric acid; phenylacetic acid; thiamine; V41; 1,2-ethanediamine; oleic acid; eicosanoyl-CoA; quinoline; icosenoyl-CoA; propinol adenylate; 2-pentadecanone; methylhexadecanoic acid; isoquinoline; acrylamide; acetyl-CoA; tyrosinamide; and phenylethylamine were increased in the feces of sows that were fed the HF diet. The levels of 2-methylhexacosane, lactate, and thiamine pyrophosphate, an active form of thiamine were increased in the feces of the MF group. The levels of propionic acid and benzofuran were significantly increased in the LF treatment compared with the HF treatment. Based upon the change in metabolites’ concentrations, metabolic pathways’ analysis identified that the HF diet mainly influenced the pyruvate metabolism; citrate cycle metabolism; glyoxylate; dicarboxylate; and thiamine metabolism compared with the LF treatment (Figure 6a). The comparison between the HF and MF treatments showed the change in pyruvate metabolism (Figure 6b).

### 2.5. Short-Chain Fatty Acid Content

The concentration of acetate in fecal samples was increased (*p* < 0.05) in sows fed the HF diet (Table 4) compared with the LF. There was no difference in fecal acetate between the MF and LF or HF treatments. The HF diet did not affect the propionate and butyrate concentration in the feces. Total SCFA (*p* < 0.05) content was increased in the HF and MF diets compared with the LF diet, however, no difference in total SCFA was observed between the MF and HF treatments.

### 2.6. Gene Expression

**Figure 7:**
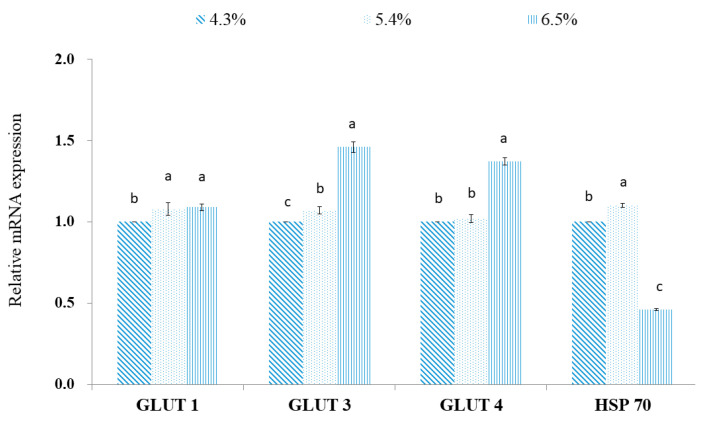
Effect of different acid detergent fiber levels on relative mRNA expression of glucose transport protein (GLUT) and heat shock protein (HSP) 70 in the placenta of sows. ^a–c^ Means with different superscripts in the same row differ significantly (*p* < 0.05).

Dietary ADF increased the gene expression of glucose transporter (GLUT)1 in the placenta of sows fed the MF and HF diets (Figure 7), however, no difference was observed between the HF and MF treatments. The gene expression of GLUT3 and GLUT4 were increased (*p* < 0.01) in the HF treatments compared with the MF and LF treatments. The gene expression of GLUT3 was increased (*p* < 0.05) in the MF treatment compared with the LF, however, no difference in gene expression of GLUT4 was observed between the LF and MF treatments. The gene expression of heat shock protein-70 (HSP) was decreased (*p* < 0.01) in the HF treatment. Moreover, a higher gene expression of HSP70 was observed in the MF treatment compared with the LF (*p* < 0.05).

## 3. Discussion

In this study, the diets showed no effects on the BW and backfat thickness during gestation and lactation. In agreement, several studies have reported no improvement in sow weight change when high fiber levels were supplemented in gestation diets [3,7]. Investigations on the influence of diet types during the transition period are limited, although gestating sows may have a different nutrient requirement regarding environmental factors and physiological adjustments. In the current study, the HF diets had relatively higher fiber and oil content, compared with the LF diets. It has already been suggested that the fibrous diet is a possible solution to increase sows’ productivity during the transition period [11,18]. The intestinal motility was increased during the periparturient period when fiber-based diets were fed to gestating sows, thereby the risk of constipation decreased during the hours before farrowing [13,19]. Zhuo et al. [20] reported an increase in fecal score by increasing dietary fiber levels in gestating sows. However, these results are mostly achieved during normal environmental conditions and there is still a stereotype to avoid using fiber during heat stress due to its higher heat increment by increasing intestinal movements. However, the result of our study shows that the addition of fiber to the diet is a necessity even during heat stress because of a significantly lower farrowing duration in sows fed the HF diet. Gestating sows are cared for in order to have a relatively shorter farrowing duration because of the serious effect on piglets’ survivability of a long farrowing duration [6,16]. Acknowledging the significance of diet type, improved nutritional strategy in the transition period to decrease farrowing duration can be considered as a potential alternative to increase sow productivity during stressful periods. The duration of farrowing is not only important for the sow but also for the piglets because of critical physical impact, which can lead to the death of the piglets [4]. Prolonged farrowing duration is an exhausting and painful period for sows, that imposes stress on piglets with ultimately an increase in the number of stillbirths [5]. Therefore, farrowing duration is a determinant factor to evaluate the quality of diet or management. This involves greater survivability of piglets, and more live-born piglets during lactation to ensure the capability of piglets in accessing the udder as soon as possible after birth [21]. However, several factors including genetics; BW; litter size; constipation index; and parity affect farrowing duration [22,23], and it is recommended that the farrowing duration should not normally be prolonged over than 4 h [12]. In the current study, the average farrowing duration (4.84 h) was higher than recommended values, possibly because of the adverse effects of heat stress. The positive effects of the HF diets on farrowing duration could be because of a higher constipation index. Practically, the feed intake of gestating sows is restricted during the last two days before the expected farrowing [17,24]. In this situation, the probability of constipation occurring increases around farrowing, due to low feed intake and fiber intake [12,25]. The high water-holding capacity of fiber increases the water content of feces and results in easing the defecation process [26]. In agreement, Shang et al. [27] showed that the inclusion of 30% wheat bran or 20% of beet pulp in the gestation diet increased the softness of feces at farrowing. Moreover, they concluded that the inclusion of 15% wheat bran or 10% of beet pulp increased fecal water content in lactating sows. In addition, it was reported that increasing the content of crude fiber in the diet of the sow from 3.8% to 7% increased water consumption and intestine motility around farrowing and thus decreased the constipation risk [19]. Moreover, a longer farrowing duration would worsen the uterine involution and possibly increase the need for a repeat of insemination during the estrus period [28]. However, the result of the current study did not show any relationship between farrowing duration and the weaning to estrus interval. Therefore, including low starch and high ADF in the diet of gestating sows is highly recommended during high ambient temperatures.

In the current study, there was no ADF effect on litter size. The use of a bulky diet for a period of two months improved the litter growth performance during the first week after farrowing [29]. In addition, the high fiber-based diet during the last three weeks of pregnancy increased the litter weight gain until day five of lactation [19]. The growth and development of mammary glands mainly occurs at the late gestation period from 80 to 115 days [11,30]. During the last third of pregnancy, the secretion of endocrine hormones markedly increases the proliferation of epithelial secretory tissues in the alveoli [11,30]. Therefore, the greater litter weight at weaning may be associated with the increase of lobular-alveolar number at late gestation, which possibly increases the milk yield in the lactation period. The treatments did not have any effect on the digestibility of DM, GE, and NDF, however, the digestibility of ADF was improved in the HF treatment. The insoluble fraction of fibers is composed of cellulose and lignin that mainly remained unhydrolyzed in exposure to endogenous enzymes during the digestion process [3,10]. Therefore, the fibrous diets increase the transit time, consequently increasing digestion and absorption duration. Interestingly, besides the increase of ADF digestibility in the HF treatment, the CP digestibility was reduced when compared with the LF- and MF-treated sows. Lowell et al. [11] reported a similar result, that high dietary fiber decreased the digestibility of protein and increased NDF and ADF digestibility.

In mammals, blood cortisol is a common indicator of physiological stress [1]. The pregnant animals are under exposure to several stressors including fetus growth; hormonal change; metabolic impact; and immunological change, which increases the blood cortisol level compared with non-pregnant sows [16]. Besides blood cortisol, the respiratory rate also could be considered as a measure of heat stress during high ambient temperature [8]. In the current study, the respiratory rate was significantly lower in the HF rather than the LF during day 98 to 104 of pregnancy, illustrating lower stress in the HF treatment. The average respiratory rate of sows reached to 71.6 mov/min on day 114 of gestation, which is an indicator of severe heat stress [1]. In addition, the lower cortisol level in the HF treatment was also in agreement with the report of Huang et al. [7] who reported reducing blood cortisol levels as the dietary fiber level increased. However, we did not evaluate blood cortisol, the hair cortisol can be a much proper indicator of chronic heat stress because of the accumulation of cortisol in the hair shaft over a long-term period. Moreover, hair sampling is a non-invasive method compared with blood sampling, particularly during a stressful period. The inclusion of 7.5% crude fiber (CF) in gestating sows’ diet decreased the cortisol concentration in saliva and stool compared with sows fed 2.5% CF [22]. There is a lack of reports on levels of hair cortisol in gestating sows, but several references indicate that hair or wool cortisol is correlated to chronic stress in cattle [22,31] and sheep [32,33]. The mechanisms underlying the influences of dietary fiber on the stress level of gestating sows have not yet been clarified, however, satiety could be associated with lower cortisol. Therefore, dietary ADF can reduce the stress level of pregnant sows during heat stress.

The fiber supplementation and its fermentation in the colon increases SCFA production including acetate, butyrate, and propionate [3,9,13]. Soluble fiber provides substrates for fermentation in the colon, which has been reported to increase SCFAs production instead of protein-based metabolites including ammonia; indole; cresol; phenol compounds; and hydrogen sulfide [15,34]. Metabolomics’ profiling of fecal samples showed differences between the HF and LF treatment. The multivariate analyses showed that the HF and LF treatments had the most obvious separations among the treatments. The VIP scores were generated to evaluate the metabolites’ contribution among different ADF levels in experimental groups. Twenty-five fecal metabolites were altered in the sows fed the HF diet. Thiamine metabolism, sulfur metabolism, and citrate cycle metabolism were perturbed in the LF treatment compared to the HF. The results showed that the vitamin B group metabolism was improved in the fecal samples of the HF sows. Thiamine pyrophosphate is the phosphorylated form of vitamin B1 with a significant role in nervous system development and maintenance [35]. The activity of the glyoxalase system can be compromised when the thiamine concentration decrease [36]. The limitation of thiamine availability reduces the proliferation of essential microbes including B. thetaiotaomicron, which belongs to the Bacteroidetes phylum and Bacteroides genus [37]. In addition, there must be a link between thiamine metabolism and thyroid hormone, a necessary hormone for optimal body metabolism, production [38], which may increase sows’ performance during heat stress. However, the immune status of sows was not evaluated in the current study, the fecal phthalic acid concentration was greater in the HF treatment, which can be an indicator of intestinal health and high immunity. There is a positive correlation between fecal phthalic acid content and IgA; IgM; IgY; IL-4; and IL-10 production [39]. In the current study, we found that the supplementation of high ADF increased the nutrient absorption and metabolism pathways including pyruvate metabolism, citrate cycle, propionate metabolism, and glyoxylate and dicarboxylate metabolism. These pathways play an important role in energy extraction and provision, targeting substrate sensing and carbohydrate substrate transporting. In addition, the level of metabolites involved in stress, such as sulfurous acid, increased in a high fiber diet, which indicated an improved antioxidant status mainly because sulfurous acid converts to sulfate to protect cells from oxidative stress [7]. The 1-Butylamine is a colorless liquid with an amine group, which has an ammonia-like odor [40]. The increase of 1-Butylamine in the feces of sows fed a high ADF diet may be associated with lower protein digestibility, resulting in more undigested protein flowing to distal sections of the gastrointestinal tract.

A low dietary starch supply during late gestation increased the total SCFA production, which possibly was due to high fiber content and fermentable carbohydrate as substrates of SCFA production at the distal part of the intestine [3,13]. Acetate, propionate, and butyrate are the main end product of the fermentation process in the large intestine [15]. There are several factors including the environment, diet, and gut microbiota to determine the amount of SCFA production [9]. It has been reported that dietary fiber is a more important substrate for SCFA production rather than dietary starch [15]. In the literature, the positive relationship between dietary fiber and fecal SCFA was confirmed [13,22,41]. However, SCFA can be produced in all sections of the gastrointestinal tract, and the colon and cecum are the main sites of SCFA production [10]. There is a hypothesis that dietary fiber can encompass carbohydrates and increase their passage to reach the large intestine and ultimately increase fermentation [42]. The most important effects of SCFA are expected to occur on carbohydrate homeostasis, lipid metabolism, immune system, and gastrointestinal health [10,22]. Short-chain fatty acids bind to specific receptors in the intestine including the G-protein-coupled receptor 41 and G-protein-coupled receptor 43 to restrict histone deacetylation and have a positive role in body metabolism [43].

The expressions of GLUT1, GLUT3, and GLUT 4 are mainly involved in the capacity of glucose transfer to the fetus resulting in improved fetal development by increasing transplacental nutrient transportation in sows [44]. The labyrinth and junction zone are the main locations for GLUT, and the expression of GLUTs, particularly GLUT3, are essential to maintain the metabolic requirements of the placenta and maternal–fetal glucose transfer [45]. Moreover, GLUT4 functions to increase the upregulation of transport in the placenta in response to fetal or maternal insulin stimulus [46]. The higher gene expression of GLUT3 and GLUT4 in the placenta indicates the greater capacity of glucose transfer, the main energy source for the fetus, to the fetus [44,46]. In the current study, the higher gene expression of GLUT3 and GLUT4 in the HF treatment did not affect fetal growth. The underlying reason may be due to the lower dietary starch in the HF diet (26%) compared with the LF diet (52%), which possibly decreases the total amount of glucose absorption. In agreement, it was reported that the downregulation of the GLUT4 gene in the placenta is a natural reaction during high blood glucose levels to protect the fetus from the adverse effects of high glucose delivery [47]. The glucose availability might be the possible underlying mechanism to explain the differences in gene expression of GLUTs. Therefore, the capacity and efficiency of GLUTs in the placenta of sows in the LF treatment had to be increased to compensate for the lower availability of blood glucose to support an adequate amount of glucose for fetal growth.

## 4. Materials and Methods

### 4.1. Experimental Design

**Figure 8:**
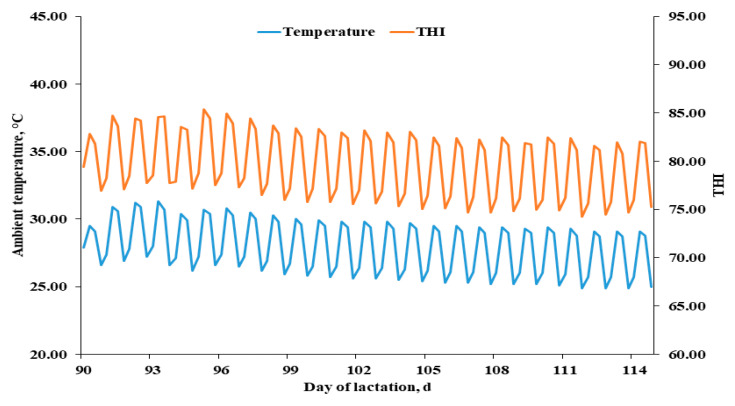
Ambient temperature (blue line) and temperature-humidity index (THI; orange line) during experimental period.

The experiment was conducted in two summer months of July–August in 2020. The management protocol was according to Kim et al. [42]. In brief, artificial insemination was performed two times after the onset of estrus, and detection of pregnancy was confirmed at day 30 post-breeding using a Pharvision B-mode ultrasound machine (AV 2100 V; Ambisea Tech. Corp, Shenzhen, China). During gestation, all sows were housed in individual gestation stalls (2.05 × 1.08 m) with fully slatted concrete flooring. All sows were moved to farrowing crates (2.14 × 2.15 m) on day 112 of gestation. Each crate had a single feeder, and water was always available through a nipple drinker. Room temperature and relative humidity were recorded every 5 min using Tenmars temperature/humidity data logger (TM-305U, Tenmars Electronics Co., Neihu Taiwan) located at a sow’s head level. The loggers recorded temperature with an accuracy of ±0.4 °C and a resolution of 0.01 °C. The humidity was recorded with an accuracy of ±3% and a resolution of 0.2%. The temperature and humidity index (THI) was calculated according to the following equation: THI = temperature −[0.55 − (0.0055 × humidity)] × (temperature − 14.5). The gestation and farrowing room temperatures are shown in Figure 8. Heating pads for piglets were located on either side of the farrowing crates and maintained at 36 °C. Lactating sows had ad libitum access to water via a drinker located in the feed trough in each farrowing crate. The feeders were checked three times per day to be refilled when required. Respiratory rates were calculated by counting the rate of flank movement in a 60-s period and then defined as breaths per minute once daily at 13:00 according to Ghassemi Nejad and Sung [48].

Thirty-six multiparous crossbred sows at day 90 of gestation (Landrace × Yorkshire; average initial BW, 191.6 ± 21 kg) were selected based on parities (18 sows in parity three and 18 sows in parity four) and BW. Sows were divided into two blocks (parity three and four) and distributed evenly between three treatments (12 sows/treatment) on day 90 of gestation. The diets included 4.3% (LF), 5.4% (MF), and 6.5% (HF) ADF in the corn-soybean-based diets. Unconsumed feed was weighed daily to determine actual feed intake. All the sows were fed a common corn-soybean meal-based diet as per NRC [49] recommendation. All gestation diets contained 3150 kcal/kg of ME, 14% CP, 0.82% Ca, 0.59% P, and 0.58% standardized ileal digestible (SID) lysine. The crude fiber content was 3.52%, 4.87%, and 6.22%; the NDF content was 12.7%, 17.7%, and 22.7%; total fiber content was 13.2%, 19.5%, and 25.9%; soluble fiber content was 1.37%, 1.88%, and 2.39%; insoluble fiber content was 11.8%, 17.65%, and 23.5%; insoluble to soluble fiber ratio was 8.62, 9.39, and 9.83 for the LF, MF, and HF diets, respectively. All sows were fed 2.5 kg of diet daily. In the lactation period, sows were fed a diet that contained 3300 kcal/kg of ME, 17.8% CP, and 0.88% SID lysine. Starting from the day after farrowing, the ration was gradually increased by one kg per day until the maximum ration was reached (2 kg + 0.6 kg per piglet) at about seven days post-partum.

### 4.2. Body Weight and Litter Performance

Bodyweight was measured on days 90 and 112 (pre-farrowing) of gestation and day 24 of lactation (weaning) as followed by Kim et al. [6]. Backfat thickness was measured at day 90 and 112 of gestation, and at day 24 of lactation at the 10th rib using an ultrasonic device (Agroscan A16, Angouleme, France). Changes in backfat thickness of sows during lactation were estimated by calculating the difference between backfat thicknesses at day 112 of gestation and backfat thickness at day 24 of lactation. Standard litter traits such as number born and born alive, BW (kg) at birth and weaning, and the numbers weaned were recorded. Feed intake (kg/d) of each sow and weaning-to-estrus interval (d) were also recorded. The value of average daily gain of piglets was calculated by final BW minus the first BW divided by weaning day multiplied by the number of weaned piglets.

### 4.3. Nutrient Digestibility

Chromic oxide (0.25%) was added into each diet from day 104 to 112 of gestation as an inert indigestible indicator to measure the apparent total tract digestibility (ATTD) of nutrients. Fecal samples were harvested from the floor during the last four days of gestation to measure the ATTD of DM; GE; CP; NDF; and ADF. The samples were mixed within the pen and dried in a forced-air drying oven at 60 °C for 72 h, and ground in a Wiley mill (Thomas Model 4 Wiley Mill, Thomas Scientific, Swedesboro, NJ, USA) using a 1-mm screen and used for chemical analysis. Experimental diets and excreta samples were analyzed in triplicate for DM (Method 930.15), CP (Method 990.03), and ADF (Method 973.18) according to AOAC [50]. The gross energy of the diets and feces was measured by a bomb calorimeter (Model 1261, Parr Instrument Co., Moline, IL), and chromium concentrations were determined with an automated spectrophotometer (Jasco V-650, Jasco Corp., Tokyo, Japan) according to the procedure of Fenton and Fenton [51]. This is an improved method for chromic oxide determination in feed and feces. The NDF was determined gravimetrically with exposure of samples to neutral detergent, amylase, and sodium sulfite, then filtration of samples on a 1.5-µm glass filter.

### 4.4. Hair Cortisol and Blood Glucose and Insulin

Hair cortisol determination was performed as described previously [52]. In brief, hair samples were shaved from the forehead of sows at days of 90 and 110 of gestation. The collected hair samples were preserved in aluminum foil and placed in polypropylene tubes to be dried (HM Hyundai Micro Co., Seoul, Korea). The samples were washed three times with isopropyl alcohol (5) mL to remove contaminations, then dried at room temperature (23 ± 1 °C) for 7 d. After drying, cortisol extraction was performed by methanol dilution to be analyzed via ELISA kit according to instructions (Cayman Chemical, Ann Arbor, MI; intra-assay CV = 8.11%, inter-assay CV = 9.3%). On 110 of gestation, ear vein catheter was subjected to collect blood samples (10) mL via catheter from all the selected sows before the morning feeding at 6:00 h at 30-min intervals for 4:30 h from 06:00 to 10:30 using a non-anticoagulant disposable tube (Becton Dickinson, Franklin, NJ, USA). Serum samples were separated by centrifugation (3000× *g* for 15 min at 4 °C), then stored at −20 °C for blood glucose and insulin analysis. A commercial kit (Fujifilm Corp., Saitama, Japan; intra-assay CV = 2.61%, inter-assay CV = 3.45%) was used for glucose determination by an automated chemistry analyzer (Fuji Dri-chem 3500i; Fujifilm Corp.). An ELISA kit (Endocrine Technologies Inc., New York, CA, USA; CV = 5.65%, inter-assay CV = 7.13%) was used to measure blood insulin concentration using an ELISA device (Biolog MicroStation system., Hayward, CA, USA).

### 4.5. Metabolomics Sample Preparation and Analysis

The metabolites concentrations were evaluated with GC-MS in sows fecal samples. In accordance with He et al. [1], 100 mg of fresh fecal sample was transferred to 5- mLcentrifuge tubes, mixed with 500 µL distilled water, and was vortexed for 60 s. Then, 1000 μL methanol was added to be an internal quantitative standard and vortexed for 30 s. The ultrasound machine was used to hold samples at 25 °C for 10 min, then the centrifuge process (5000 r/min; 5 °C; 15 min) was performed after 30 min incubation on ice. All the supernatants were placed in 2 mL centrifuge tubes and dried. Then, the dried samples were mixed with 60 μL of methoxyamine solution in pyridine and vortexed (30 s) to be reacted for 120 min at 37 °C. A 60 μL trifluoroacetamide reagent (containing 1% FMCS) was added for 90 min (37 °C) and centrifuged (5000 r/min; 5 °C; 15 min). The produced supernatant was moved to a sample bottle to be analyzed by Agilent 7890A/5975C GC-MS (Agilent Technologies, Santa Clara, CA, USA).

### 4.6. Short Chain Fatty Acids

On day 112 of gestation, a fresh fecal sample was collected by rectum massage and stored at −80 °C until the end of the collection period, then immediately transferred to the laboratory. In accordance with Mohammadigheisar et al. [53], approximately 1 g of feces was grabbed, weighed, and diluted with 2 mL of deionized water, then to obtain a supernatant the sample was centrifuged at 10,000× *g* (4 °C) for 20 min. Next, a ratio of 9:1 (sample: 25% of metaphosphoric acid solution) was mixed and centrifuged at 3000× *g* for 10 min. The supernatant was aspirated with a syringe and filtered through a 0.45 mm filter membrane. Acetate, propionate, butyrate, and total SCFA were evaluated using gas chromatography (YL 6500 GC, Gyeonggi-do, Korea; TRB-G43 capillary column with 30 m length and inner diameter of 0.53 mm, and film thickness of 3 µm), equipped with a flame ionization detector. Column temperature started at 70 °C and increased to 150 °C after 3 min. Injector and detector temperature was 250 °C, and each injection volume was 1 µL.

### 4.7. Gene Expressions

The total RNA of placenta tissue was extracted using Trizol (Invitrogen, Carlsbad, CA, USA), and quantified at an absorbance ratio of 260/280 with the Thermo Scientific™ μDrop™ Plate (Thermo Scientific, Waltham, MA, USA) and Multiscan GO (Thermo Scientific, Waltham, MA, USA). The 500 ng of total RNA was reverse transcribed to complementary DNA (cDNA) using the Improm-II Reverse transcription system (Promega, Fitchburg, MA, USA). Polymerase chain reaction (PCR) condition and selection of primers were conducted following the description by Hosseindoust et al. [54], and Gao et al. [55]. Reverse transcription-quantitative real-time PCR (RT-qPCR) was conducted using the Real-Time System (Mx3000P, Stratagen, La Jolla, CA, USA). The RT-qPCR primers including HSP70, GLUT1, GLUT3, GLUT4, and reference gene (β-actin) were designed (Table 5). The relative mRNA expression levels of β-actin as a housekeeping gene was used for normalizing gene expression. The relative fold change of mRNA was determined using 2−ΔΔCT method.

### 4.8. Statistical Analyses

The statistical analysis was performed using the GLM procedure (SAS Inst. Inc. Cary, NC). The statistical model used in this study included ADF levels and parity number as fixed effects and initial BW as a covariate. Treatment means were separated by Tukey multiple range tests. An individual sow was used as an experimental unit for analysis of all variables. Probability values of ≤0.05 were considered significant. For metabolites’ analysis, the collected raw data were analyzed and the metabolites were detected (http://srdata.nist.gov/gateway/, accessed on 6 February 2022) and normalized to (13C2)-myristic acid and stable isotope IS. The statistical analysis was performed with the SIMCA-P+ version 13.0 software package (Umetrics, Umea, Sweden). The VIP values of 1.0 and *p*-values of 0.05 were considered as metabolites that could be evaluated between three ADF treatments. The impact of heat stress on metabolic pathways and metabolite set enrichment analysis were determined according to the online tool (http://www.metaboanalyst.ca/faces/ModuleView.xhtml, accessed on 6 February 2022) [1].

## 5. Conclusions

In conclusion, the results of the present study show that the inclusion of 6.5% dietary ADF could decrease the adverse effects of heat stress during the gestation period. Several metabolites were increased in fecal samples with an increase in dietary fiber. However, more research is needed to evaluate the role of dietary ADF on metabolites’ production and pathways in different stages of heat stress. Further, the carry-over effects of 6.5% total ADF inclusion during the gestation period were reflected in the lactation period by improving feed consumption, reproductive performance, and litter growth. To our knowledge, this study is the first to evaluate the effects of total dietary ADF during heat stress and its carry-over effects on the lactation period.

## Figures and Tables

**Table 1:** Effect of dietary acid detergent fiber (ADF) levels in gestation diets on sows’ performance.

ADF (%)	4.3	5.4	6.5	SEM	*p*-Value
BW, kg					
Day 90	192.22	190.44	192.33	1.17	0.775
Day 112	206.89	206.11	205.52	0.91	0.832
Weaning	169.44	171.00	169.94	1.05	0.830
Loss during Lactation	37.45	35.11	35.58	0.73	0.376
BF, mm					
Day 90	21.05	20.46	20.17	0.24	0.336
Day 112	21.35	20.75	20.49	1.03	0.163
Weaning	16.24	16.19	16.14	0.19	0.982
Loss during lactation	5.11	4.59	4.35	4.68	0.456
ADFI kg/d					
During lactation	4.77 ^b^	5.05 ^ab^	5.25 ^a^	0.07	0.008
Farrowing duration, h	5.24 ^a^	4.92 ^b^	4.45 ^c^	0.12	<0.001
Constipation index ^1^	1.20 ^c^	1.80 ^b^	2.63 ^a^	0.08	<0.001
Weaning to estrus interval, day	5.25	5.15	5.11	0.19	0.826

^1^ A score value ranging from 0 to 5: 0, absence of feces; 1, dry and pellet-shaped; 2, between dry and normal; 3, normal and soft, but firm and well-formed; 4, between normal and wet; still formed, but not firm; and 5, very wet feces, unformed and liquid. ^a,b,c^ means with different superscripts in the same row differ significantly (*p* < 0.05). SEM, standard error of means; BW, body weight; BF, backfat thickness; ADFI, average daily feed intake.

**Table 2:** Effect of dietary acid detergent fiber (ADF) in gestation diets on litter performance.

ADF (%)	4.3	5.4	6.5	SEM	*p*-Value
Litter size					
Total born	12.70	12.40	12.50	0.15	0.721
Weaned	10.30	10.20	10.50	0.21	0.849
Mortality	2.40	2.20	2.00	0.13	0.325
Piglet weight, kg					
At birth	1.29	1.31	1.27	0.04	0.937
At weaning	5.77 ^b^	5.89 ^ab^	5.98 ^a^	0.03	0.045
Litter weight, kg					
At birth	14.80	15.07	14.30	0.59	0.874
At weaning	56.99 ^b^	59.64 ^ab^	60.79 ^a^	0.65	0.043

^a,b^ means with different superscripts in the same row differ significantly (*p* < 0.05). SEM, standard error of means.

**Table 3:** Effect of dietary acid detergent fiber (ADF) levels in gestation diets on apparent total digestibility (%) of nutrients.

ADF (%)	4.3	5.4	6.5	SEM	*p*-Value
DM	88.4	88.63	87.27	0.61	0.682
GE	89.33	88.37	87.5	0.59	0.506
CP	88.87 ^a^	85.57 ^b^	81.17 ^c^	1.16	0.001
NDF	64.07	64.73	65.37	0.58	0.714
ADF	55.27 ^c^	58.13 ^b^	60.77 ^a^	0.85	0.002

^a–c^ Means with different superscripts in the same row differ significantly (*p* < 0.05). SEM, standard error of means; DM, dry matter; GE, gross energy; CP, crude protein; NDF, neutral detergent fiber; ADF, acid detergent fiber.

**Table 4:** Effect of dietary acid detergent fiber (ADF) levels in gestation diets on fecal SCFAs concentration (d 112).

ADF (%)	4.3	5.4	6.5	SEM	*p*-Value
Acetate (μmol/g)	69.56 ^b^	71.68 ^ab^	72.67 ^a^	0.53	0.041
Propionate (μmol/g)	16.8	17.3	17.9	0.43	0.577
Butyrate (μmol/g)	7.52	7.41	7.54	0.06	0.725
Total SCFAs (μmol/g)	93.85 ^b^	96.40 ^a^	98.56 ^a^	0.61	0.003

^a,b^ Means with different superscripts in the same row differ significantly (*p* < 0.05). SEM, standard error of means; SCFA, short-chain fatty acids.

**Table 5:** Primers used for real-time PCR analyzing.

Gene	Nucleotide Sequence of Primers (5′–3′)
GLUT1	F: GCAGGAGATGAAGGAGGAGAGC
	R: ACGAACAGCGACACGACAGT
GLUT3	F: GCCCTGAAAGTCCTCGGTTCCT
	R: ACACGGCGTTGATGCCAGAGA
GLUT4	F: GGCCATCGTCATTGGCATTC
	R: GTCAGGCGCTTCAGACTCTT
HSP70	F: GCCCTGAATCCGCAGAATA
	R: TCCCCACGGTAGGAAACG

HSP, heat shock protein; GLUT, glucose transporter.

## Data Availability

The data presented in this study are available on request from the corresponding author. The data are not publicly available due to the further study required.
